# Radiographic Assessment of Anatomic Risk Factors Associated with Acute, Lateral Patellar Dislocation in the Immature Knee

**DOI:** 10.3390/sports4020024

**Published:** 2016-04-15

**Authors:** Thai Trinh, Andrew Mundy, Matthew Beran, Kevin Klingele

**Affiliations:** 1 Mount Carmel Health Systems, Department of Orthopedics 793 West State Street Columbus, Columbus, OH 43222, USA; thai.trinh.12@gmail.com; 2 Ohio State University Medical Center, Department of Orthopaedics, 410 W 10th Ave, Columbus, OH 43210, USA; andrew.mundy@osumc.edu; 3 Nationwide Children’s Hospital, Department of Orthopedics, 700 Children’s Drive, Ste. A2630, Columbus, OH 43205-2696, USA; matthew.beran@nationwidechildrens.org

**Keywords:** children, knee, magnetic resonance imaging, morphology, patella, patellofemoral joint

## Abstract

Acute patellar dislocation remains a common injury in both adult and pediatric patients. Non-operative management has been advocated for patients without a history of recurrent instability. Although pathologic thresholds for consideration of operative management have previously been reported in adults, it is largely unknown in children. A retrospective review of all skeletally immature patients diagnosed with acute lateral patellar dislocation who had MRI imaging were included for analysis. An age-based control group was also identified. Six radiographic measurements were compared: lateral trochlear inclination (LTI), trochlear facet asymmetry (TFA), trochlear depth (TD), tibial tuberosity–trochlear groove (TT–TG), sulcus angle (SA) and patellar height ratio. A total of 178 patients were included for analysis (study: *n* = 108, control: *n* = 70). The mean age of patients in the study and control groups was 13.7 and 12.1 years respectively (*p* ≤ 0.001). Study group patients had significant differences in all radiographic measurements including a decreased LTI (*p* < 0.001), increased TFA (*p* < 0.001) and SA (*p* < 0.001). The mean trochlear depth was 3.4 mm and 5.6 mm for patients in the study and control groups respectively (*p* < 0.001). Study group patients had an increased patellar height ratio (*p* < 0.001) and TT–TG distance (*p* < 0.001). Morphologic abnormalities may predispose skeletally immature patients to an increased risk of acute lateral patellar instability.

## 1. Introduction

Acute patellar dislocation represents 2%–3% of all knee injuries in adults and children [1,2,3,4,5]. Traditionally, management of patients following primary patellar dislocation has been non-operative, characterized by a short period of immobilization followed by physiotherapy and gradual return to activities [6,7]. Recurrent patellar instability has previously been reported in up to 60% of patients following non-operative management, and has prompted some authors to advocate early surgical intervention aimed at reducing the morbidity associated with recurrent instability, including the time lost from activity and the development of early-onset patellofemoral arthritis [4,8,9,10,11,12,13].

Radiographic assessment of anatomic risk factors for patellar dislocation has traditionally included the use of plain radiographs and computed tomography (CT) [6,7]. More recently, magnetic resonance imaging (MRI) has become an important diagnostic tool in the evaluation of patients with patellofemoral instability. Although surgical management of recurrent patellar instability continues to evolve, current techniques aim to correct the anatomic risk factors that appear to predispose patients to patellar instability [14,15]. Previous studies have identified morphologic differences between skeletally mature patients with and without patellar instability. Radiographic thresholds that can aid in operative planning have been established based on anatomic differences [9,14,16,17]. It is largely unknown if these thresholds are applicable to skeletally immature patients, as there are only a limited number of studies reporting radiographic norms in pediatric patients with patellar instability [15,18] The purpose of the current study is to report morphological differences between skeletally immature patients with and without acute, lateral patellar instability. 

## 2. Methods

Following the institutional review board (IRB) approval, a retrospective review (August 2009–June 2014) of patients diagnosed with an acute patellar dislocation was performed. Inclusion criteria consisted of skeletally immature patients that had a diagnosis of acute patellar dislocation and had an MRI of the symptomatic knee. The diagnosis of acute patellar dislocation was made clinically with a history and/or physical examination consistent with patellar dislocation. Exclusion criteria included skeletal maturity (defined as closed distal femur physis on plain radiograph and/or MRI), patients with obligatory or habitual patellar instability, and patients who had undergone a previous knee surgery altering their native anatomy (e.g., tibial tubercle transfer, trochleoplasty). A total of 108 patients (108 knees), all between the ages of 9 and 16 years old, were identified. All patients enrolled were skeletally immature with open physes on imaging. 

To obtain the control group, a retrospective review of skeletally immature patients presenting between 2002 and 2014, who had undergone MRI imaging to evaluate for knee pathology other than patellar dislocation, was performed. Only MRIs interpreted by a pediatric-trained radiologist as normal or without pathologic features interfering with normal morphology (e.g., popliteal cysts, discoid menisci, and small effusions) were included for analysis. Ten patients were randomly selected at every age within the age range of our acute patellar dislocation study group (9–16 years old) for comparison. This identified a total of 70 patients (70 knees) for analysis (control group).

**Figure 1:**
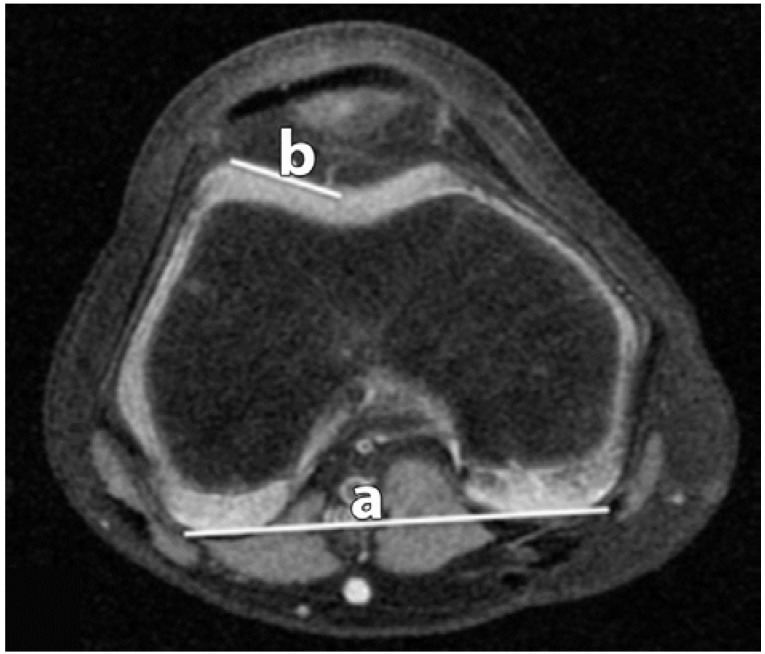
Lateral Trochlear Inclination (LTI). The angle between the lateral trochlear facet (**b**); and the line along the posterior condyles (**a**) is measured.

**Figure 2:**
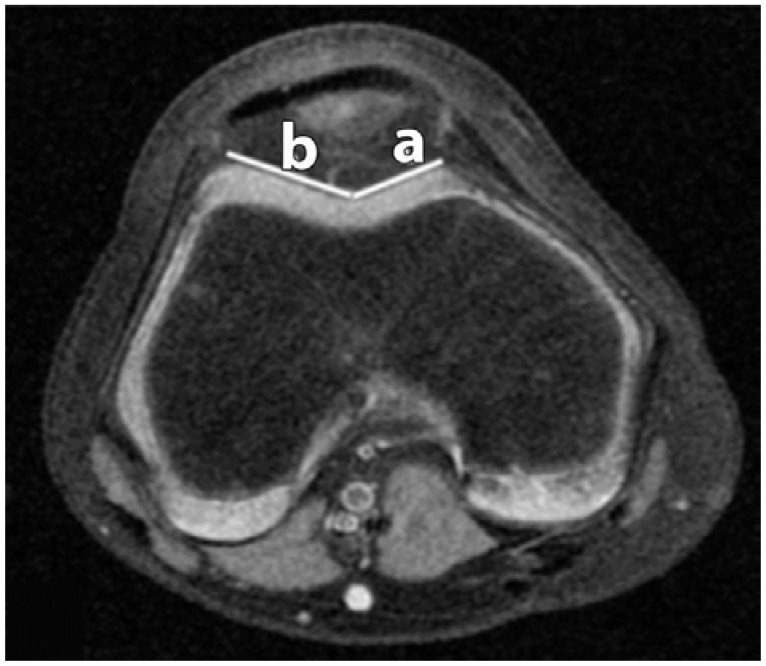
Trochlear Facet Asymmetry (TFA). The distances of the lateral facet (**b**); and medial facet (**a**) are recorded.

**Figure 3:**
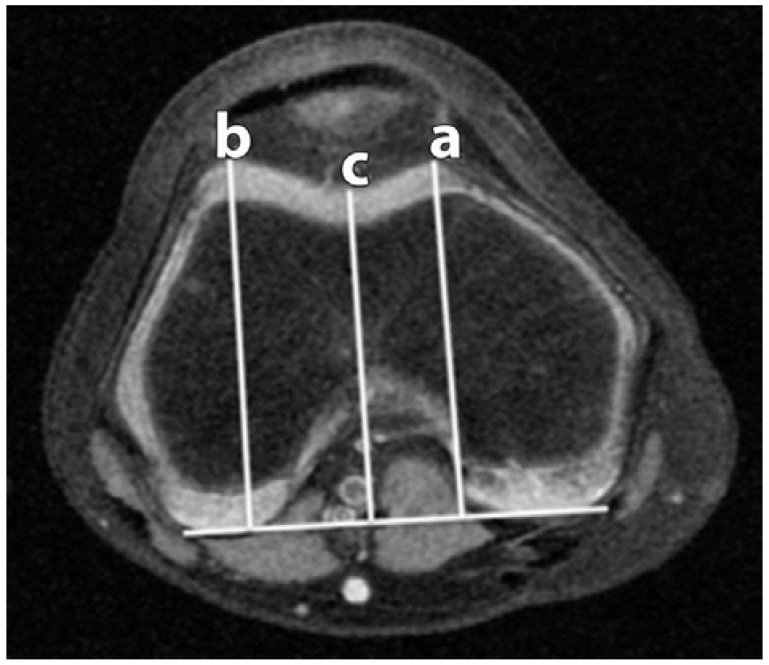
Trochlear Depth (TD). A posterior condylar reference line is created (solid white line). Three lines are drawn from the reference line to the lateral facet apex (**b**); medial facet apex (**a**); and the deepest portion of the sulcus (**c**).

**Figure 4:**
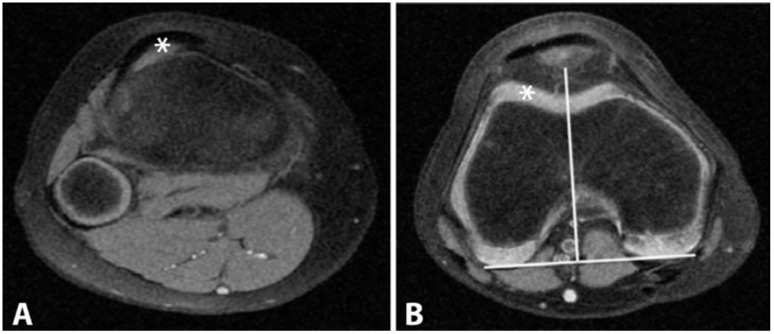
(**A**) and (**B**): Tibial Tuberosity–Trochlear Groove (TT–TG) distance. The superior attachment of the patellar tendon at the tibial tuberosity is marked (**A**); and then transposed on our standard axial sequence (**B**). The distance between the marker (*) and solid white line that is extending through the deep sulcus to the reference line was recorded.

**Figure 5:**
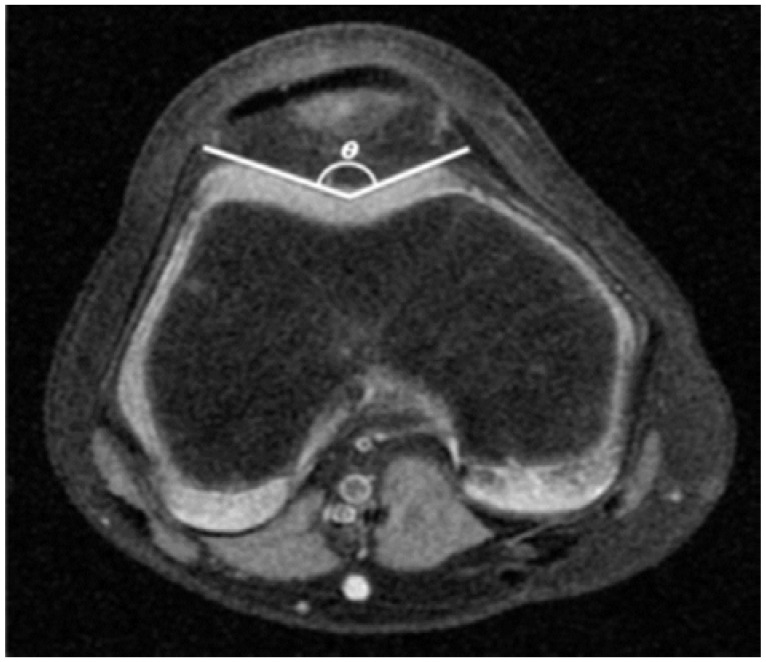
Sulcus Angle (SA). The angle (θ) between the lateral and medial facet is measured.

**Figure 6:**
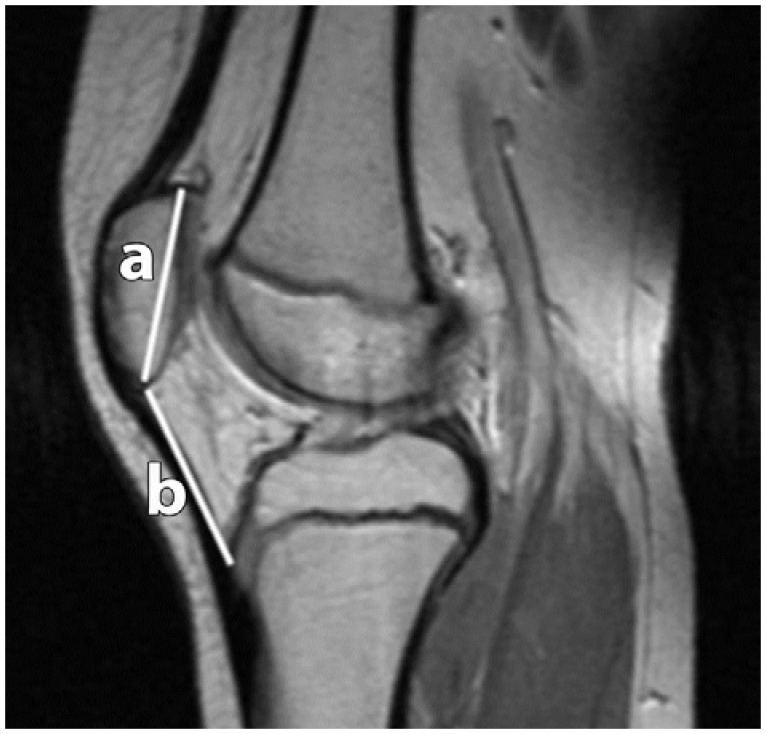
Patellar Height Ratio (PHR) according to Insall-Salvati. Two measurements were recorded, the distance between the superior apex (articular surface) and inferior apex (non-articular surface) (**a**); and the distance of the patellar tendon at its attachments from the inferior patellar apex to the tibial tuberosity (**b**).

MRI images were obtained at our institution utilizing MRI scanners possessing a magnetic field strength ranging from 1.5 to 3.0 T. A total of six measurements were recorded utilizing either T2 or proton density (PD) sequences: lateral trochlear inclination (LTI), trochlear facet asymmetry (TFA), trochlear depth (TD), tibial tuberosity–trochlear groove (TT–TG), sulcus angle (SA) and patellar height ratio according to Insall-Salvati [17] (Figure 1, Figure 2, Figure 3, Figure 4, Figure 5 and Figure 6).

These measurements were selected based on their use in previous literature assessing patellofemoral morphology [5,17].

Statistical analysis was performed utilizing the StatPlus LE software (AnalystSoft, Walnut, CA, USA) [19]. All continuous data satisfied the assumption of normality and thus only parametric analysis was performed. Continuous outcome data between study and control groups were analyzed utilizing a two-sample T test. Categorical data between groups were analyzed utilizing a Pearson Chi-square test. Statistical significance was set at *p* < 0.05.

## 3. Results

A total of 178 patients were included for analysis (study: *n* = 108, control: *n* = 70). The mean age for patients in the study and control groups was 13.7 and 12.1 years, respectively (*p* < 0.001). There was no difference in the distribution of males and females (*p* = 0.29) or laterality (*p* = 0.55) between groups (Table 1).

**Figure 7:**
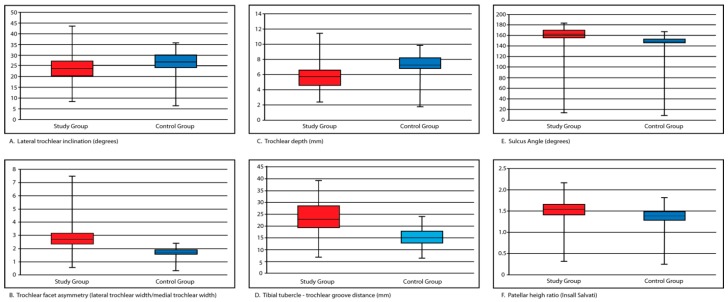
Distribution of lateral trochlear inclination (**A**); trochlear facet asymmetry (**B**); trochlear depth (**C**); tibial tubercle—trochlear groove distance (**D**); sulcus angle (**E**); and patellar height (**F**) between study and control groups.

All radiographic measurements of interest were obtained in patients comprising the study and control groups. Summarized data is presented in Figure 7.

Measurements characterizing the morphology of the trochlea (LTI, TFA, TD, and SA) were statistically different between patients in the study and control groups (Table 2).

Patients in the study group were characterized as having a decreased lateral trochlear inclination (15.6° *vs.* 20.7°, *p* < 0.001), increased sulcus angle (150.2° *vs.* 141.6°, *p* < 0.001) and an increased trochlear facet asymmetry (2.3 *vs.* 1.5, *p* < 0.001). The study group also demonstrated a shallower trochlear groove compared to patients in the control group (3.4 mm *vs.* 5.6 mm, *p* ≤ 0.001). The mean TT–TG distance for patients in the study and control groups were 17.0 mm and 8.9 mm, respectively (*p* < 0.001). A difference in patellar height was also noted between groups with patients in the study group (1.3 ± 0.2 mm) possessing increased patellar height compared to controls (1.1 ± 0.2 mm) (*p* < 0.001).

## 4. Conclusions

The purpose of the current study was to examine morphological differences between skeletally immature patients with and without acute, lateral patellar instability. Our findings suggest that anatomic differences in trochlear and patellar morphology may result in an increased risk of acute patellofemoral instability in skeletally immature patients. Previous studies examining patellofemoral instability in adults have suggested that surgical correction of aberrant anatomy may decrease the risk of recurrent instability. Although pathologic thresholds for considering surgical treatment in adults have been proposed [7,12,20], it is unclear if these same thresholds are appropriate for the skeletally immature patient, as normal radiographic parameters used to quantify patellofemoral morphology have yet to be established in this population [15]. Mundy *et al*.[18] have shown high inter and intraobserver reliability in the utilization of such measurements in the immature knee. In addition, such measurements develop with maturation, gradually reaching adult norms by age 10 [15,18].

In a retrospective analysis of patients with and without patellar instability, Charles *et al.* [14] reported significant variations in trochlear morphology using MRI. Although the mean age and the status of the distal femoral/proximal tibial physis of patients examined in this study were not disclosed, patients experiencing recurrent patellar instability were shown to have significant differences in trochlear morphology as assessed by the lateral trochlear inclination, trochlear facet asymmetry, trochlear depth, and sulcus angle. The current study reports similar findings in a subset of skeletally immature patients.

Despite the identified differences in trochlear morphology between skeletally mature and immature patients, the clinical implications as it relates to operative management are currently undefined. Previous literature has reported satisfactory outcomes following trochleoplasty in skeletally mature patients experiencing recurrent instability in the setting of trochlear dysplasia [21]. There is likely a limited role for this procedure in the pediatric population due to implications associated with physeal arrest. Furthermore, little is known about the post-operative changes in patellofemoral kinematics and its influence on cartilaginous and bony development following a trochleoplasty procedure. Similarly, tibial tubercle transfers cannot be performed until proximal tibial physeal closure has occurred. Stabilization procedures in the skeletally immature knee thus rely on soft tissue procedures which do not disturb growth. 

Limitations of the current study are primarily related to its retrospective design. In order to identify patients for the control group, we retrospectively analyzed all patients who underwent MRI imaging of the knee without a history of patellofemoral instability. In doing so, there is inherent selection bias for patients who presented for evaluation of knee pathology. Differences may exist between this group and those asymptomatic patients who would not have undergone MRI evaluation. Additional limitations include the wide age range of patients in both the study and control groups. For the purposes of the current study, patients were categorized as skeletally immature based on the presence of an open distal femoral physis. There is currently a paucity of literature pertaining to age related changes of the patellofemoral joint. It is possible that different stages of development may alter the anatomy in such a way that what would be considered pathologic at one stage of development, but falls into the spectrum of normal during another. Therefore, considering skeletally immature patients as a group makes the assumption that irregular anatomy contributes to the development of patellofemoral instability equally throughout development. Larger studies are needed to further evaluate the possibility of age related changes to the patellofemoral joint and the implications of these changes as it pertains to management of patients with patellar instability.

Despite these limitations, the current study is the first to compare radiographic differences in patellofemoral morphology between skeletally immature patients with and without acute, lateral patellofemoral instability. Results suggest morphologic abnormalities may predispose skeletally immature patients to an increased risk of such injury. Further research is needed to determine the clinical implications of these differences in order to optimize both surgical and non-surgical management of recurrent instability in skeletally immature patients.

## Figures and Tables

**Table 1:** Selected patient demographics.

Variables	All (*n*)	Study (*n*)	Control (*n*)	*p*
Patients	178	108	70	–
Age (Years)	13.1 ± 1.9	13.7 ± 1.42	12.1 ± 2.1	<0.001
Sex				0.29
Male	93	53	40	
Female	85	55	30	
Side				0.55
Left	104	65	39	
Right	74	43	31	

**Table 2:** Summarized anatomic differences between patients with and without patellar instability utilizing MRI.

Measured Variables	Study Mean ± SD	Control Mean ± SD	*p*
Lateral trochlear inclination (degrees)	15.6 ± 5.5	20.7 ± 3.9	**< 0.001**
Trochlear facet asymmetry (%)	2.3 ± 0.8	1.5 ± 0.3	**< 0.001**
Trochlear depth (mm)	3.4 ± 1.5	5.6 ± 1/1	**< 0.001**
Tibial tubercle trochlear groove distance (mm)	17.0 ± 6.7	8.9 ± 4.1	**< 0.001**
Sulcus angle (degrees)	150.2 ± 10.1	141.6 ± 6.1	**< 0.001**
Patellar height (%)	1.3 ± 0.2	1.1 ± 0.2	**< 0.001**
